# Quantitative systems-based prediction of antimicrobial resistance evolution

**DOI:** 10.1038/s41540-023-00304-6

**Published:** 2023-09-07

**Authors:** Daniel A. Charlebois

**Affiliations:** 1 Department of Physics, University of Alberta, Edmonton, AB T6G-2E1 Canada; 2 Department of Biological Sciences, University of Alberta, Edmonton, AB T6G-2E9 Canada

**Keywords:** Evolution, Biological physics

## Abstract

Predicting evolution is a fundamental problem in biology with practical implications for treating antimicrobial resistance, which is a complex system-level phenomenon. In this perspective article, we explore the limits of predicting antimicrobial resistance evolution, quantitatively define the predictability and repeatability of microevolutionary processes, and speculate on how these quantities vary across temporal, biological, and complexity scales. The opportunities and challenges for predicting antimicrobial resistance in the context of systems biology are also discussed. Based on recent research, we conclude that the evolution of antimicrobial resistance can be predicted using a systems biology approach integrating quantitative models with multiscale data from microbial evolution experiments.

## Introduction

Predicting evolution is an important fundamental and practical problem in biology and medicine. The ability to quantitatively predict evolution will advance evolution from a descriptive theory to a predictive theory^1,2^ that can tackle global health problems such as *antimicrobial resistance* (AMR)^3^ (Box 1). The rapid evolution of pathogens in response to antimicrobial drugs motivates the need to transform evolution into a predictive, quantitative science to develop “evolution proof” drugs^4^ and vaccines^5^, which will respectively enable effective treatments and prophylactics to combat AMR.

There is a pressing need to develop a predictive theory of AMR evolution to answer numerous outstanding questions. First, what do *evolutionary predictability* and *evolutionary repeatability* mean in the context of AMR evolution and which aspects of resistance can be forecasted for microbes? These questions are challenging given the multiple scales at which AMR occurs, ranging from the rapid emergence of resistance mutations inside of cells^6,7^ to the longer-term establishment of complex resistance-conferring dynamics among microbial communities^2^, along with the fact that AMR is a complex system-level evolutionary process^8^. Next, can we predict evolutionary paths (trajectories) or are we restricted to evolutionary endpoints (outcomes)? Distinguishing between trajectories and outcomes is essential since evolutionary processes can follow different trajectories to arrive at the same evolutionary outcome or they can take similar trajectories but diverge to yield different evolutionary outcomes^9^. Last, which variables are important to develop predictive models of *microevolution*? This is critical for model development as well as the design of microbial evolution experiments, which can generate high-dimensional data (e.g., genetic sequence) or low-dimensional data (e.g., phenotypic growth rate).

Throughout this article, we tackle the questions posed above and explore the challenges and opportunities for predicting AMR evolution from a systems biology perspective, which incorporates the complex interactions between different scales and components of evolving microbial systems. We conclude that the evolution of AMR can be predicted (see “Resistance evolution across the scales” for examples) using quantitative systems-based models informed by multiscale data from high-replicate or high-temporal resolution experiments on evolving microbial systems.

Box 1 Terminology *Antimicrobial Resistance (AMR)*: AMR arises when bacteria, fungi, viruses, parasites, or other microbes no longer respond to antimicrobial drugs.

*Clade*: A group of genetically related organisms that evolved from a common ancestor.

*Clonal Interference:* Competition between beneficial mutations in different lineages with fixed genetic backgrounds in asexual populations.

*Deterministic*: Describes a process that evolves along the same trajectory to the same future state from a given initial state.

*Epistasis*: The phenotypic expression of a genetic mutation/gene is dependent on the expression of one or more other mutations/genes.

*Evolutionary Predictability*: A statement about the evolutionary trajectory/future state of a biological system, defined by the existence of a probability distribution (see “Limits on predicting antimicrobial resistance” for more details).

*Evolutionary Repeatability*: How likely a particular evolutionary event is to repeatedly occur in a biological system, defined by the likelihood, entropy, or other measures of variability (see “Limits on predicting antimicrobial resistance” for more details).

*Fitness*: The reproductive success of an organism. Growth rates are often used as a proxy for fitness in microbes.

*Fitness Landscape*: A visual representation of the relationship between genotype and fitness.

*Genetic AMR*: Drug resistance that is mediated by genetic mutations or through the acquisition of drug resistance genes.

*Genetic Drift*: The change in gene variant (allele) frequency in a population due to random chance.

*Macroevolution*: Large evolutionary changes such as the emergence of a novel pathogen over long timescales.

*Microevolution*: Change in allele frequency over a short timescale (as compared to macroevolution).

*Nongenetic AMR*: Drug resistance that arises among genetically identical microbes exposed to the same drug, which is not the result of genetic AMR.

*Stochastic*: Describes a process that randomly evolves along one of many possible trajectories to one of many possible future states from a given initial state.

### Predictability versus repeatability in microbial evolution

**Fig. 1:**
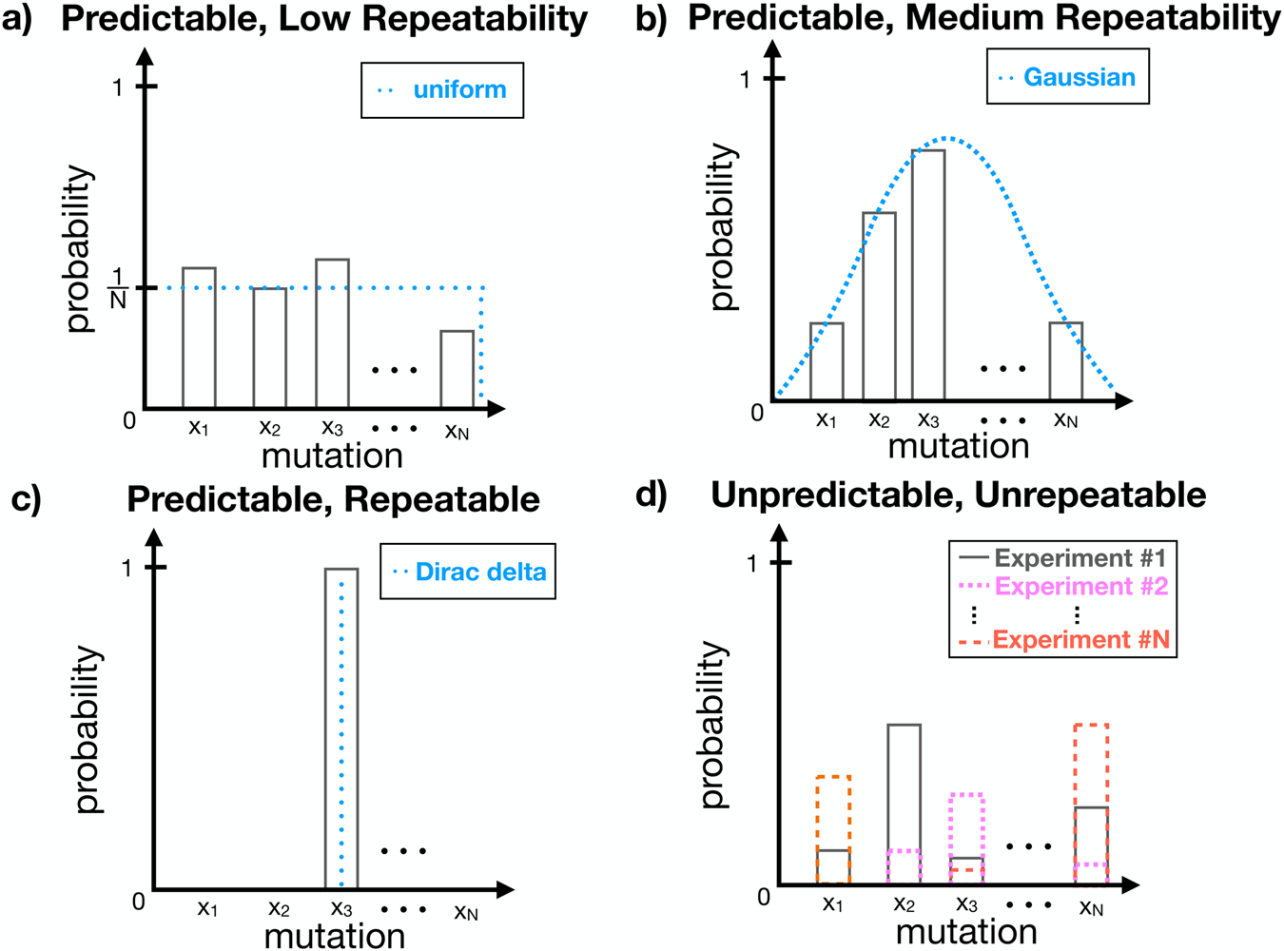
Predictability versus repeatability in antimicrobial resistance evolution. Schematic of the probability of drug resistance mutations occurring during an evolution experiment or patient treatment. **a** Predictable: described by a uniform distribution. Low repeatability: maximum entropy (${H}_{\max }=\log N$). **b** Predictable: described by a Gaussian distribution. Medium repeatability: medium entropy ($H=\frac{1}{2}({{\log }}\left(2\pi {\sigma }^{2}\right)+1)$, where ${\sigma }^{2}$ is the variance). **c** Predictable: described by a Dirac delta distribution (here $\delta \left({x}_{k}\right)=1$ and $\delta \left({x}_{i\ne k}\right)=0$). High repeatability: minimum entropy $({H}_{\min }={{\log }}(1))$, as the same mutation emerges for every experiment or treatment. **d** Unpredictable: no distribution can be derived or fit empirically to the data. Unrepeatable: different outcomes for each experiment or treatment.

Researchers lack a shared language to describe evolution^2^. We propose that evolving biological microbial systems be quantified based on their *evolutionary predictability* and their *evolutionary repeatability*. The predictability of an evolutionary process is ultimately a probabilistic statement about a biological system (or ensemble), which can be defined by the existence of a probability distribution (Fig. 1). If a probability distribution can be derived theoretically or obtained empirically, then an evolutionary process can be statistically predicted. For instance, if there is a known distribution of outcomes (e.g., of resistance mutations) for a given antimicrobial drug applied during an AMR evolution experiment or patient treatment, then the ensemble behavior of this system is predictable. Predictive distributions that change over time as biological systems evolve require a “dynamic” (time-dependent) model, as opposed to unchanging predictive distributions that can be described by a “steady-state” (time-independent) model^10^. However, the existence of a predictive distribution does not guarantee that any particular mutation (or other resistance traits such as gene expression level^11^) will be easily repeated or realized in an evolution experiment or during patient treatment. An analogy can be drawn to picking cards from a deck of playing cards; while the distribution of outcomes is predictable, picking a specific card is not easily repeatable.

Evolutionary repeatability is related to the likelihood of occurrence of individual events that constitute a statistical ensemble. Repeatability can be quantified using measures from statistical physics such as entropy (Fig. 1). If an evolutionary trajectory or outcome is highly uncertain, stochastic, or entropic, then it has low repeatability. This will reduce the realization of a specific trajectory or outcome in a microbial evolution experiment or during the treatment of a patient, such as a particular biological replicate or patient acquiring a specific sequence of drug resistance mutations. Shannon entropy^12^

1 $$H=E\left[-{{\log }}\left(p\left(x\right)\right)\right]=-\mathop{\sum }\limits_{i=1}^{N}{p}_{i}\log ({p}_{i})\;{\rm{or}}-{\mathop{\int}\nolimits_{\!-{\infty}}^{\infty}}p\left(x\right){\log}\left(p\left(x\right)\right){dx}$$

or other measures of variability can be used to quantify repeatability. Note that the entropy associated with a deck of cards is higher than that of a dice, due to the higher dimensionality (i.e., more equally probable outcomes) of the deck of cards compared to the dice. Here, if we consider an ensemble of drug resistance mutations that may appear during an evolution experiment or patient treatment, a uniform distribution represents maximum uncertainty or entropy (Fig. 1a), a Dirac delta function minimum uncertainty (Fig. 1c), and other distributions (e.g., Gaussian) intermediate uncertainty (Fig. 1b) in predictable evolutionary processes. An evolutionary process may also be unpredictable (no distribution describes it) and unrepeatable (the state space of possible outcomes changes each time) (Fig. 1d). AMR evolution is repeatable when different pathogen populations evolve similar resistance to an antimicrobial drug (e.g., acquired mutations in the same genes of the same pathogen infecting different patients)^13^ and larger selection pressures are known to generate more repeatable evolution^14,15^. While AMR repeatability has been observed for some large-effect sequence changes^16–18^, it has been more broadly observed for molecular resistance phenotypes^19,20^.

### Limits on predicting antimicrobial resistance

**Fig. 2:**
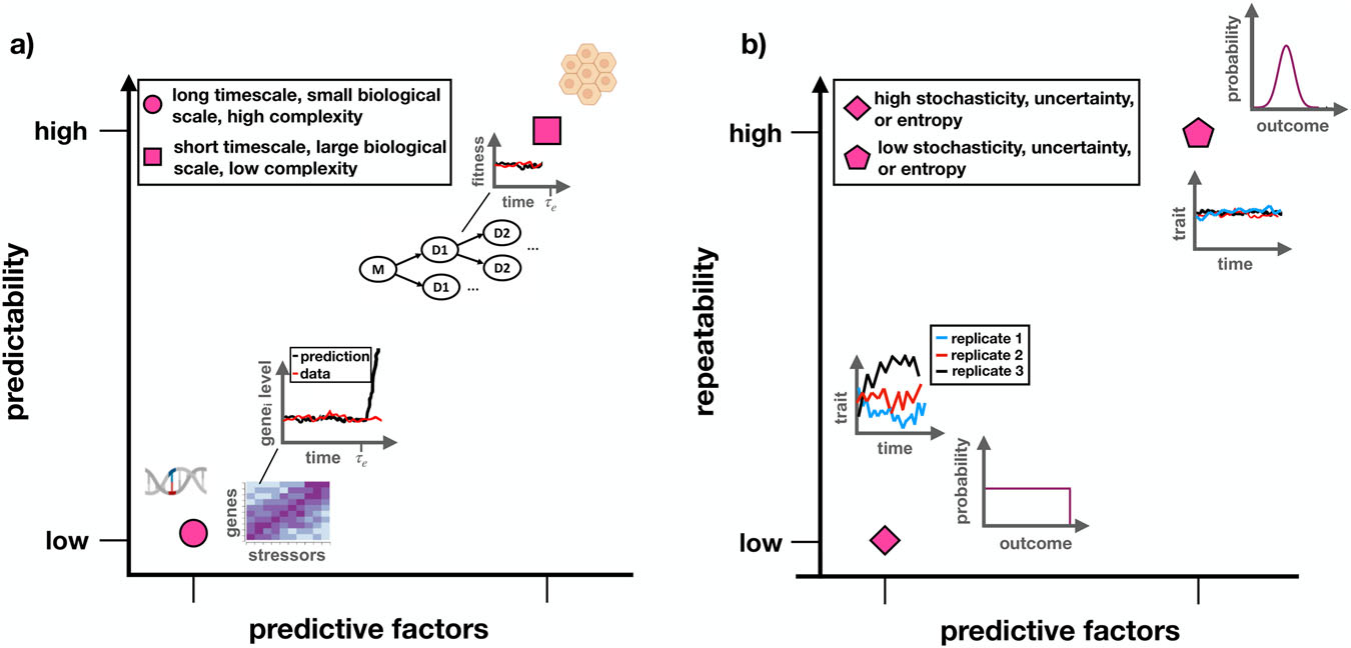
Limits on evolutionary predictability and repeatability. **a** The predictability of microevolution decreases at long timescales (e.g., longer than the generation time of a microorganism), small biological scales (e.g., single-nucleotide polymorphisms), and high dimensionality/complexity (e.g., genomic profiles), whereas the predictability of microevolution increases at short timescales (e.g., shorter than the generation time of a microorganism), large biological scales (e.g., whole pathogen populations), and low dimensionality (e.g., phenotypic fitness measurements). ${\tau }_{e}$ is the characteristic time for predicting evolution beyond which predictions become random. **b** The repeatability of evolution decreases as the stochasticity of evolutionary trajectories/outcomes increases, or as the uncertainty/entropy of the associated probability distribution increases. This schematic was made in part using BioRender.com (2023).

There are fundamental and practical limitations to predicting AMR evolution (Fig. 2). Predictability is fundamentally constrained by random genome mutations and *genetic drift*^21^. This requires that a *stochastic* framework^22^ be applied to analyze and make predictions based on data obtained from measurements of biological quantities (e.g., mutations, growth rates, etc.) at low concentrations during early stages or at late stages (if drug treatment is effective) of microbial evolution experiments and infections; genetic drift also plays a confounding role in this regime^9^. Recently, stochastic systems-based population models were proposed to guide drug therapies by providing predictions on resistance mutation appearance probabilities and first-appearance times, indicating timescales for substituting or combining antimicrobial drug during patient treatment^23^. Random genetic drift distorts the impact of selective forces and decreases the ability of models to predict evolution^2^.

Other sources of biological stochasticity also pose a challenge for predicting the evolutionary path to resistance, including gene expression variability among genetically identical cells^22,24^ and genetic drift^25^. The interplay between *nongenetic AMR* and *genetic AMR* is not fully understood^26^, and such knowledge gaps negatively impact our ability to predict resistance evolution. It has been hypothesized that nongenetic AMR facilitates evolution by allowing a fraction of the cell population to survive and subsequently evolve genetic resistance to antimicrobial treatment^11,27^. Systems-based stochastic population dynamics modeling has shed light on the nongenetic-genetic AMR enigma by incorporating resource competition between nongenetically resistant and genetically resistant subpopulations; these models predict that nongenetic resistance facilitates survival but slows down genetic AMR evolution^23^. Similar models can be inferred from coupled stochastic trajectories of gene expression and cell division^28^. The architecture of systems of interconnected genes comprising genetic networks modulates the amplitude and timescale of gene expression fluctuations, which enhances acute, reversible, nongenetic drug resistance^11,29,30^ and facilitates prolonged, nonreversible, genetic drug resistance^6,26^.

The interactions and competition among mutations, along with data limitations, are other important considerations for predicting AMR evolution. *Epistasis* arises from a non-additive interaction between mutations that affect *fitness*^31^. Mutations that arise early in the evolution of AMR affect which mutations are subsequently selected^32^. Epistatic interactions generate a nonlinear *fitness landscape*^33^. This occurs because whether a mutation is beneficial, detrimental, or neutral in terms of selection depends on the genetic, extracellular, and environmental background^1,34^. Beneficial mutations may synergistically interact to decrease selective pressure and render evolution slower and more stochastic (since weaker selection is less *deterministic*), thus reducing repeatability. For instance, beneficial mutations in disjoint *clades* compete for fixation, whereas mutations in nested clades reinforce one another in *clonal interference*, a phenomenon that arises in large asexual microbial populations subject to strong selective pressures such as antimicrobial drug therapy^34,35^. Clonal interference may enhance predictability by reducing stochastic waiting times for fitter genetic variants and by ensuring that beneficial mutations that fix have a large fitness effect and are driven by natural selection, not by genetic drift or environmental noise^1^. Clonal interference was found to be pervasive in an experimental model of *E. coli* in the mammalian intestine, and the targets of natural selection were similar in independently evolving bacterial populations, resulting in similar early-stage phenotypic changes^36^. Another fundamental challenge in predicting AMR evolution lies in the unknowns, complexities, and redundancies in mapping genotype to phenotype. Our ability to predict AMR evolution on fitness landscapes with considerable epistasis is highly dependent on the precision of the initial conditions data^9^. Epistasis can make predictive modeling more challenging, as the order of and interactions between mutations must be accounted for as well as the fact that epistasis facilitates the evolution of novel functions^37^; on the other hand, epistasis can constrain evolutionary trajectories to a given endpoint thus potentially increasing repeatability. Despite these challenges, metabolic fitness landscapes have been utilized to predict antibiotic resistance^38^. Data limitations further hinder our ability to predict resistance evolution, such as uncertainty in initial conditions for chaotic environmental systems in which microorganisms evolve^39^, even for evolution driven by deterministic natural selection^9^.

It has been argued that biology cannot be reduced to physics, as the complexity of living organisms and the biosphere is too vast to predict how life will evolve^40^. Others have argued that physics can be used to predict evolution on shorter timescales (Fig. 2)^9,41^. Evidence now exists supporting the predictability of AMR microevolution. For instance, in yeast mutations that emerged during evolution experiments on cells harboring synthetic drug resistance gene networks were computationally predicted beforehand based on the costs and benefits of expressing the synthetic gene network in a particular drug condition^7^. In bacteria, the same set of resistance mutations have been found to repeatedly fix in independently evolving populations^18^, suggesting that antibiotic resistance evolution is predictable and repeatable. Antiviral resistance mutations have also been predicted for SARS-CoV-2 using in silico mutational scanning inhibitor docking^42^. However, it is unlikely that *macroevolution* can be predicted for practical as well as fundamental reasons. Similar to the Lyapunov time (i.e., the characteristic timescale beyond which the chaotic trajectory of a dynamical system can no longer be predicted^43^), there exists a characteristic timescale for predicting evolving biological systems (${\tau }_{e}$) due to stochasticity in the data and errors in the predictive model^1^. Fluctuating selective pressures can also decrease repeatability due to the evolution on continuously changing fitness landscapes; the selection timescale for beneficial mutations to emerge is reduced and the outcome of selection becomes more divergent^44^.

### Resistance evolution across the scales

Evolutionary predictability and repeatability vary across temporal, biological, and complexity scales (Fig. 2). Evolution has been shown to be more convergent at “higher” biological scales (e.g., the acquisition of drug resistance genes during treatment) than at “lower” biological scales (e.g., individual resistance mutations within a gene)^45^. For instance, the majority of single-nucleotide and amino acid changes occur in a single population in laboratory evolution experiments^45–47^ (i.e., different populations follow divergent paths in sequence space^1^). Additionally, patients respond to an infection or vaccination by different immune receptor sequences^48^. However, even at the genetic scale notable examples exist where microevolution is highly repeatable, such as the establishment of the same mutation due to extensive standing variation^49^ or strong selective pressure^50^.

AMR resistance evolution is more predictable when the dimensionality of the state space is reduced. For instance, the state space of all genotypes is high-dimensional, whereas the state space of fitness is one dimensional. Even despite the constraints imposed by the number of paths by selection, genome evolution is generally unpredictable as sequence space contains an effectively infinite number of possible evolutionary paths^1^. On the other hand, AMR evolution is more predictable than general genome evolution due to the finite number of AMR mutations and resistance mechanisms^51^. Phenotype space (e.g., fitness^46,52^) is also more predictable, as different mutations can have similar effects on fitness (i.e., different sequence changes map onto the same phenotypic effects). However, analyzing low-dimensional traits may be less informative than high-dimension traits, as low-dimensional traits are expected to yield a lower information gain relative to a prior, which can be quantified as the log ratio of predicted probability (*q(t)*) to prior probability (*p*(*t*)): ($I(t)={\rm{\log }}(q(t)/p(t))$)^53^. Though few mutations have been found to be shared between replicates in high-replicate bacterial evolution experiments, adaptive convergence has been shown to emerge at higher levels of biological organization, including genes, operons, and functional complexes^45,54^.

An important consideration for predicting AMR evolution is that pathogens evolve within large, highly complex and interconnected ecosystems that are themselves evolving^55^. It remains unclear how ecological diversity in these biosystems, such as multiple pathogenic yeast species co-infecting a patient^56^, or diversity-generating processes such as negative frequency-dependent selection (which favors rare phenotypes over common ones)^9,57^, affect the predictability of AMR evolution. As the complexity of ecological interactions increase, adaptive tradeoffs to multiple selective pressures reduce our ability to predict evolution^58,59^. Though ecological-evolutionary feedbacks can increase our ability to predict evolution through negative frequency-dependent selection^60^.

### Opportunities and challenges for systems-based prediction of antimicrobial resistance

Machine learning is not yet prevalent in evolutionary studies, although it shows promise for predicting AMR. For instance, machine learning models have been trained to predict the evolutionary success of complex systems, such as the human influenza virus variants^61^. However, at present, machine learning models largely function as context-specific “black boxes” that do not elucidate the mechanisms underlying AMR evolution^51^, though this situation may change depending on progress made in of the field of explainable artificial intelligence^62^. In contrast, quantitative systems-based models coupled with evolution experiments on pathogens with well-cataloged resistance mutations would serve as powerful tools for elucidating the mechanisms underlying AMR evolution. It remains to be seen if quantitative systems-based modeling will possess the same predictive power as machine learning.

The performance of quantitative systems-based models trained on data generated from computationally or microbial evolution experiments can be evaluated using information measures^63,64^. This approach may prove valuable in identifying the characteristic time for predicting evolution (Fig. 2) over which evolutionary dynamics can be predicted by quantitative and machine learning models. Testing the predictions made by AMR evolution models in evolution experiments on microbes carrying genetically engineered drug resistance networks^65^ would be particularly useful for the iterative “test, build, deploy” cycle that is common in synthetic biology. As drug resistance genes do not function or evolve in isolation, a systems approach rather than a reductionist approach has led to new targets against AMR mediated by genetic networks that control resistance genes^6,29,30,65,66^. For instance, therapeutically targeting regulator genes as opposed to resistance genes has been predicted to mitigate AMR^65^.

A promising avenue is to take a systems-based approach to predict AMR evolution based on properties that are not exhibited by individual resistance genes or microbial species, such as the emergent properties that are associated with interacting genes or microbes^9,67^. Models of rare, random evolutionary events could be validated by performing massively parallel simulations^68^ or microbial evolution experiments with many replicates (ranging from tens to hundreds) and analyze a large collection of endpoint measurements^65^. Alternatively, researchers could perform in silico or in vitro/in vivo evolution experiments with a small number of replicates and track the evolutionary dynamics at a higher temporal resolution. Ideally, these experiments would measure evolutionary trajectories as well as endpoints at multiple scales (including growth assays, imaging, and high-throughput “omics” data generated from genomics, transcriptomics, proteomics, and metabolomics technologies). Another promising approach is to use DNA barcoding, which can detect mutations at low frequencies in cellular lineages and estimate their time of occurrence as well as fitness effects^69,70^. DNA barcoding is particularly helpful at the beginning of microbial evolution experiments when many mutations are present at low frequencies, in contrast to whole genome sequencing which is beneficial at the end of microbial evolution experiments when few resistance mutants have fixed in the population. Genome editing and deep mutational scanning open the possibility of quantifying the number AMR mutations and their individual fitness effects to predict drug resistance^71^ and guide drug development^72^. The systems biology approach of combining different levels of omics technologies with genome-scale metabolic models can provide precision and robustness to AMR predictions^8^. Finally, systems-based epidemiological models of AMR evolution that include the structure of the host population, interactions between genetic loci, and integration of within- and between-host levels can predict strategies to limit the spread of drug-resistant pathogens^73^.

## Discussion

Microbial evolution spans temporal, biological, and complexity scales, which differentially impact our ability to predict AMR. We can quantitatively define evolutionary predictability in terms of our ability to define probability distributions and evolutionary repeatability in terms of likelihood or entropy. Evolutionary predictability and repeatability can also be thought of in terms of information gain relative to a prior^64^, which is related to the entropy of the system^74^. The predictability of microevolutionary processes such as AMR increases on short timescales^41^, whereas macroevolution, such as the emergence of a new pathogen, takes place on timescales well beyond the characteristic timescale for predicting evolution. AMR evolution is anticipated to be more predictable but less repeatable at larger biological scales, while at small biological scales it is less predictable but more repeatable. The complexity of biological systems presents practical challenges for making predictions but is also imposes constraints that can increase predictability. In particular, constraints arising from competing infectious agents^23^ or from drug combinations^75^ may aid the prediction of resistance evolution.

Predicting AMR evolution is an interdisciplinary endeavor that will require combining multiple fields including physics, mathematics, computer science, and the biological sciences to achieve breakthroughs. One promising strategy is to develop predictive models of evolution using a systems-based approach^76^, which incorporates dynamics across multiple scales ranging from the interactions between genes in a genetic drug resistance network^29,30^ to species interactions in microbial communities^9^. The application of machine learning is another approach which promises to play an increasingly important role in drug design, patient diagnostics, and predicting AMR^77^. Ultimately, to predict AMR evolution we need to overcome two challenges: (1) develop quantitative, multiscale models of complex fast-resistance evolving biological systems and (2) generate high-replicate data that span time, space, and biological organization from systems-based evolution experiments to inform and test predictive models. Overcoming these challenges will enable clinicians to predict AMR evolution, revolutionizing the treatment of patients with drug-resistant infections.

### Reporting summary

Further information on research design is available in the Nature Research Reporting Summary linked to this article.

### Supplementary information

Reporting Summary

## Data Availability

Data sharing is not applicable to this article as no new data were created or analyzed in this study.
